# Pazopanib, Cabozantinib, and Vandetanib in the Treatment of Progressive Medullary Thyroid Cancer with a Special Focus on the Adverse Effects on Hypertension

**DOI:** 10.3390/ijms19103258

**Published:** 2018-10-20

**Authors:** Rikke Vilsbøll Milling, Daniela Grimm, Marcus Krüger, Jirka Grosse, Sascha Kopp, Johann Bauer, Manfred Infanger, Markus Wehland

**Affiliations:** 1 Department of Biomedicine, Aarhus University, 8000 Aarhus C, Denmark; rikke-milling@hotmail.com; 2 Clinic and Policlinic for Plastic, Aesthetic and Hand Surgery, Otto-von-Guericke-University, 39120 Magdeburg, Germany; marcus.krueger@med.ovgu.de (M.K.); sascha.kopp@med.ovgu.de (S.K.); manfred.infanger@med.ovgu.de (M.I.); markus.wehland@med.ovgu.de (M.W.); 3 Department of Nuclear Medicine, University of Regensburg, 95053 Regensburg, Germany; jirka.grosse@klinik.uni-regensburg.de; 4 Max-Planck Institute of Biochemistry, 82152 Martinsried, Germany; jbauer@biochem.mpg.de

**Keywords:** medullary thyroid carcinoma, hypertension, VEGF, antiangiogenesis, tyrosine kinase inhibitors

## Abstract

Medullary thyroid cancer (MTC) is a rare malignancy with a poor prognosis. First line therapy is surgery, which is the only curative method of the disease. However, in non-operable cases or with tumor progression and metastases, a systemic treatment is necessary. This form of cancer is often insensitive to conventional chemotherapy, but the use of tyrosine kinase inhibitors (TKIs), such as pazopanib, cabozantinib, and vandetanib, has shown promising results with an increase in progression-free survival and prolonged lifetime. Therefore, we focused on the pharmacological characteristics of TKIs, their mechanism of action, their application as a secondary treatment option for MTC, their efficacy as a cancer drug treatment, and reviewed the ongoing clinical trials. TKIs also act systemically causing various adverse events (AEs). One common AE of this treatment is hypertension, known to be associated with cardiovascular disease and can therefore potentially worsen the well-being of the treated patients. The available treatment strategies of drug-induced hypertension were discussed. The mechanism behind the development of hypertension is still unclear. Therefore, the treatment of this AE remains symptomatic. Thus, future studies are necessary to investigate the link between tumor growth inhibition and hypertension. In addition, optimized, individual treatment strategies should be implemented.

## 1. Introduction

The medullary thyroid carcinoma (MTC) occurs in two forms: sporadic (75%) and hereditary (25%), in most cases with mutations in the proto-oncogene tyrosine-protein kinase receptor gene *RET*. Upon diagnosis, the most common treatment method of the disease is surgical intervention, including total thyroidectomy and central neck dissection, given that metastatic spread to cervical lymph nodes is a common event. Surgical cure is possible, but in progressive cases of the disease and distant metastatic spread, this treatment method is not sufficient [1]. Distant metastases are present in 13–15% of cases, and occur most often in the lungs, liver, and bone. These patients have a poor prognosis. The metastatic disease-form of MTC needs a systemic treatment, but is insensitive to conventional chemotherapy, external beam radiation, and radioactive iodine therapy for thyroid cancer (TC). Therefore, a targeted therapy is needed. Tyrosine kinase inhibitors (TKIs), such as pazopanib and cabozantinib, are proposed as a promising new therapeutic option. TKIs work by blocking internal signaling cascades involved in the induction of angiogenesis, an important process for the tumor to acquire enough nutrients for further expansion [1,2]. However, such therapies also have adverse effects (AEs), and for tyrosine kinase inhibitors, two of the most common side effects are proteinuria and hypertension [3].

## 2. Thyroid Cancer

Depending on the cells of origin, several thyroid cancer types can be distinguished. The dominant form of thyroid cancer is differentiated thyroid cancer (DTC), caused by a malignant transformation of the follicular cells (FCs). There are two forms of DTC: papillary thyroid cancer (PTC) and follicular thyroid cancer (FTC) [4]. Rare forms of cancer developed in the FCs are poorly differentiated thyroid cancer (PDTC) and anaplastic thyroid cancer (ATC) [5]. When malignant transformations occur in the parafollicular cells (C cells), the resulting cancer characteristics differ in many ways from other forms of thyroid cancer, i.e., in their lack of iodine uptake. This form is known as MTC [4].

Of all thyroid malignancies, MTC accounts for about 2%, which translates into roughly 1000 new cases per year in the U.S. [6]. In Denmark, MTC accounts for 8% of all thyroid cancers, but it is still the rarest form [7]. MTC appears in two variants: a sporadic form that develops in 75% of cases, and an inherited form that is linked to a germ-line mutation of the *RET* proto-oncogene. The inherited form is divided into three subgroups, depending on the clinical manifestations and findings caused by the different mutations of the *RET* gene. They are known as multiple endocrine neoplasia (MEN) type 2A, MEN type 2B, and as familial MTC (FMTC), respectively. The *RET* gene codes for a receptor tyrosine kinase (RTK), which upon stimulation, promotes cell proliferation and differentiation [8]. Mutations in the *RET* gene are the cause in 43% of the sporadic form incidents, and their presence is believed to be associated with a more persistent disease and lower overall survival [9].

MTC is highly likely to spread to the regional lymph nodes, and more distant metastases in liver, lungs, and bones occur occasionally as a result of the rich lymphatic drainage and vascularization of the thyroid. A lymphatic spread has often already occurred by the time of diagnosis. With MTC not being sensitive to either of the non-surgical treatments (radioactive iodine therapy, external beam radiation, conventional therapy), the first line treatment is a thyroidectomy combined with lymph node dissection. Regardless, the need for sustainable systemic treatment options remains [10]. The current therapy 10-year survival rate is 76.5–80.5%, but in patients with distant metastases, this drops to 40% [11,12].

## 3. Tyrosine Kinase Inhibitors

Despite a general resistance against standard systemic anti-cancer therapy, tyrosine kinase inhibitors (TKIs) show good results as a secondary treatment option for MTC [13]. 

Tyrosine kinases (TKs), or receptor tyrosine kinases (RTKs), are a family of receptors found throughout the body. RTKs are monomers (except for the insulin-receptor), which, upon stimulation from ligands, form dimers that cause an autophosphorylation followed by a conformational change and activation of tyrosine kinases. This triggers the Ras/Raf/MAPK signaling cascade, along with possible concurrent signaling via the phosphoinositide 3-kinase (PI3K) pathway. This leads to changes in transcriptional factors and consequently changes in expression patterns of genes involved in cell growth, differentiation, and cell survival [14]. With TK being the target for growth factors (GF), TKIs are important anticancer drugs, preventing tumor growth by acting anti-angiogenically [15].

Tissue growth, such as tumor development, necessitates a constant nutrient supply, which is accomplished by the development of new blood vessels from pre-existing ones, a process termed angiogenesis. This development is regulated by multiple GFs, but most significantly by vascular endothelial growth factors (VEGFs), which stimulate vascular permeability, cell proliferation, and cell survival in both the physiological and pathophysiological angiogenesis. Other GFs also contribute to angiogenesis. Amongst these, the fibroblast GF (FGF), hepatocyte GF (HGF), and platelet derived GF (PDGF) are of most importance [16,17].

The VEGF family comprises five members (VEGF-A, VEGF-B, VEGF-C, VEGF-D, and placental GF (PGF)), which interact with their corresponding RTKs, the VEGF receptors (VEGFRs). VEGFRs can be divided into three types, and while VEGFR-1 and -2 are expressed in vascular endothelial cells, VEGFR-3 is expressed in lymphatic endothelial cells. All VEGFRs are characterized by different specificities (Table 1) [18].

Two pharmacological approaches are available to interrupt VEGF signaling. First is the direct inhibition of VEGF using monoclonal antibodies, preventing the binding to their receptors [19]. However, it has been shown that cancers are able to develop resistance towards these drugs, which in turn can lead to an increase in expression of hepatocyte growth factor receptor MET, the only known receptor for HGF. Consecutively, this leads to an increase in aggressiveness and metastatic progression of the tumor [20]. 

The second pharmacological approach to prevent tumor progression is the inhibition of the RTKs, prompting a blockage of consecutive intracellular phosphorylation cascades. This is the method of action of cabozantinib, pazopanib, and vandetanib. Cabozantinib, an orally administered TKI, is given in doses ranging from a daily dose of 0.08 mg/kg to 11.52 mg/kg and inhibits MET, VEGFR-2, and RET, thus blocking the phosphorylation cascade and thus suppressing any oncogenic effects. At the same time, the problem of a potential upregulation of MET expression due to VEGF inhibition is eliminated [21]. 

Pazopanib is another orally administered TKI, used in doses of 800 mg/d [22], inhibiting all VEGFRs, the PDGF receptor (PDGFR), and c-Kit tyrosine kinases [23]. It has a terminal elimination half-life of 91.3 h, reaches steady state plasma-levels after 15 d and is metabolized in the liver by cytochrome P_450_2C8 (CYP2C8) [24]. 

Vandetanib is one of the first TKIs tested in patients suffering from MTC and has been approved for treatment of the disease in 2011 in the U.S. and in 2012 in Europe. It is bound to plasma proteins by more than 99.9% and has a terminal eliminiation half-life of 31.1 h with a main secretion via the feces (82.2%). The mean bioavailability was found to be 21.4% [25]. Vandetanib’s main targets are RET, VEGFR-2, and epidermal growth factor receptor EGFR [24,25,26,27]. The usual dosage ranges from 100 mg/d to 300 mg/d [26,27,28,29,30,31]. Vandetanib’s pharmacokinetic properties were linear over the dosage range of 50 to 1200 mg/d. The maximum plasma concentration of 857 ng/mL is usually reached after 6 h. Vandetanib is highly protein-bound (92–94%) and has a terminal excretion half-life of 20 d. It is metabolized by cytochrome P_450_3A4 (CYP3A4) and predominantly excreted via the feces and urine (44% and 25%, respectively) [32]. 

**Figure 1:**
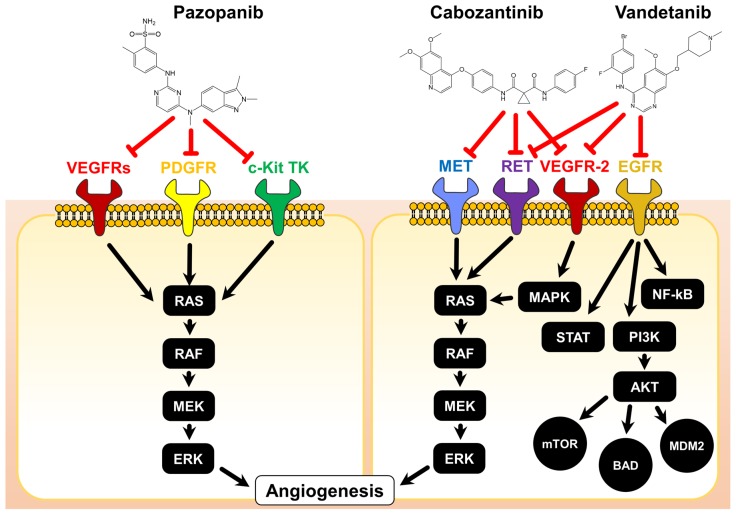
Chemical structures and modes of action of pazopanib, cabozantinib, and vandetanib. The red T-arrows indicate inhibition on receptor signaling. AKT: Protein kinase B; BAD: BCL2 associated agonist of cell death; c-Kit: stem cell growth factor receptor, EGFR: epidermal growth factor receptor; ERK: extracellular regulated kinase, MAPK: mitogen activated protein kinase, MEK: methyl ethyl ketone, MET: methionine, MDM2: mouse double minute 2, mTOR: mammalian target of rapamycin, NF-kB: nuclear factor kappa B, PDGFR: platelet derived growth factor receptor, PI3K: phosphoinositide 3 kinases, RAF: rapidly accelerated fibrosarcoma, RAS: rat sarcoma, RET: rearranged during transfection, STAT: signal transducers and activators of transcription.

The modes of actions of the three discussed TKIs are illustrated in Figure 1.

### 3.1. Adverse Effects

The inhibitory effect of VEGF signaling resulting from many TKIs, such as cabozantinib, pazopanib, or vandeatnib, is not only a local, tumor-selective effect. TKIs also act systemically and can give a rise to a multitude of different AEs, although they are generally better tolerated than other cytotoxic chemotherapeutics. Not surprisingly, among the most common AEs are those related to the cardiovascular system, such as hypertension or chronic heart failure in combination with dermatologic (skin rash, skin and hair hypopigmentation, alopecia), abnormal laboratory value (anemia, leucopenia) or more unspecific AEs such as diarrhea, nausea, fatigue, or anorexia [33,34,35,36]. While most of these AEs require a strict management to ensure patient compliance and preservation of quality of life as established for other chemotherapeutics, the cardiovascular side effects need special attention, as they are very specific to this class of drugs and potentially life-threatening when untreated. The underlying mechanisms of this drug-induced hypertension are to date not fully clarified [37]. In animal models, VEGF stimulation has been shown to result in the production of nitric oxide (NO), presumably through the PI3K pathway, leading to NO-mediated hemodynamic changes e.g., vasodilation and increased vascular permeability, thus reducing the total peripheral resistance (TPR) and decreasing the blood pressure [38]. Another animal study demonstrated that inhibition of the VEGF signaling cascade by blocking VEGFR2 via antibodies results in reduced levels of NO and the development of hypertension, thereby providing a possible explanation for the TKI-induced hypertension [37].

It was also suggested that the onset of hypertension during TKI-treatment might be connected to the reduction of capillary density. The number of small vessels is of significant importance for the TPR. A decrease leads to greater resistance and consequently to higher blood pressure. Using side stream dark field imaging, it has been shown that the number of mucosal capillaries decreased after the onset of TKI treatment. However, the study was unable to conclude whether the lack of NO or the reduced number of small vessels was the source of origin for the observed hypertension [39].

It has also been demonstrated that VEGF and endothelin-1 (ET1) mutually mediate each other’s gene expression levels, resulting in an increased protein synthesis and release. With ET1 being a potent vasoconstrictor under normal physiological conditions, and VEGF generally being a vasodilator, thereby neutralizing each other’s effect, it might be assumed that pharmacological blocking of VEGFR could lead to hypertension via overexpression of ET1 [37,40].

**Figure 2:**
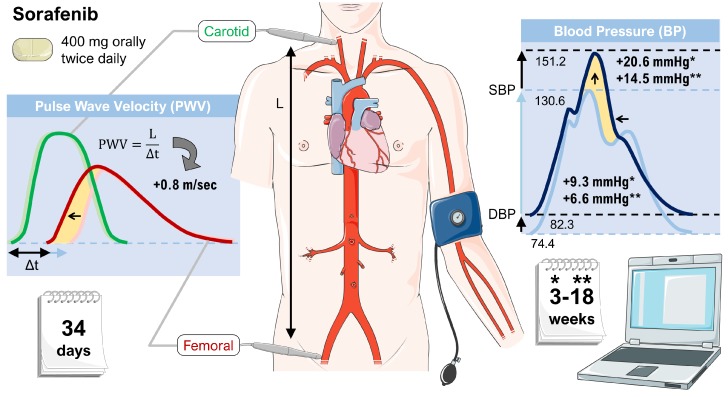
Effects of sorafenib on vascular stiffness. Patients treated with sorafenib showed an increase in systolic blood pressure (SBP) and diastolic blood pressure (DBP) as well as an elevation of the pulse wave velocity (both indicated by yellow areas). Sorafenib was administered to patients continuously at a dose of 400 mg twice daily. The data is based on a subset of patients enrolled onto a phase II randomized discontinuation clinical trial [41]. Parts of the figure were drawn by using pictures from Servier Medical Art.

Lastly, in a study investigating the TKI sorafenib using parameters such as the central aortic augmentation index (CAIx) and the aortic pulse wave velocity (APWV), treatment with TKI has shown to significantly increase aortic stiffness, suggesting that vascular stiffness could contribute to drug-induced hypertension. However, the study was not able to clarify whether the stiffness occurred as a result of hypertension or was a cause of it [41]. Figure 2 shows a graphical representation of the most important findings.

### 3.2. Efficacy of Cancer Drug Treatment

Previously, pazopanib showed promising results for the treatment of thyroid cancer, reporting effects in DTC with a 49% Response Evaluation Criteria in Solid Tumors (RECIST) partial tumor response rate (PR), but the agent had no effect in ATC. Therefore, a study on the efficacy of pazopanib in metastatic and progressive MTC was conducted, and had demonstrated promising results. The study met its pre-trial criteria for success with a RECIST tumor response rate of 14.3%, further estimated the median progression-free survival (PFS) to be 9.4 months, and the median overall survival 19.9 months, providing a considerable treatment alternative for progressive MTC [22].

A double-blind phase III trial compared the primary end point, PFS after treatment with cabozantinib compared to a placebo in the treatment of progressive MTC. This trial found PFS to be significantly longer with cabozantinib, with a median of 11.4 months compared to only 4.0 months when using placebo. The estimated tumor response rate for cabozantinib and placebo is 28% and 0%, respectively, with results varying independently on RET-status, thus making it a promising treatment option [34]. However, the difference for the secondary study end point, overall survival (OS), was found to be statistically insignificant. Despite being insignificant, patients receiving cabozantinib had a median OS of 26.6 months vs. a median OS of just 21.1 months for placebo patients (HR 0.85; 95% CI 0.64 to1.12; *p* = 0.24), resulting in an actual difference of 5.5 months. This provides patients treated with cabozantinib a 20.68% longer survival period compared to the placebo group. In addition, the study demonstrated that patients with RET M918T-positive tumors may have a greater benefit of cabozantinib than patients of the RET M918T-negative subgroup [42].

After promising phase 2 trials, which showed 20% PR plus an extra 53% of stable disease (SD) in patients suffering from unresectable, locally advanced or metastatic MTC treated with 300 mg/d vandetanib [28], 16% PR, and 53% SD under a 100 mg/d vandetanib regimen [29] in a similar patient cohort, and 47% PR in children and adolescents with locally advanced or metastatic hereditary MTC [31], a larger study was conducted. This multicenter, randomized, phase 3 trial included a total of 331 patients with locally advanced or metastatic MTC and aimed to compare efficacy and safety of 300 mg/d vandetanib (*n* = 231) with placebo (*n* = 100). It was shown that vandetanib therapy significantly prolonged PFS (30.5 months vs. 19.3 months; hazard ratio 0.46; 95% CI 0.31 to 0.69; *p* < 0.001) and resulted in 45% PRs [30]. In addition, positive effects of vandetanib were also found for objective response rate, disease control rate, and biochemical response (all *p* < 0.001). No definite statements could be made about OS, as at the time of data publication, the median follow-up period of 24 months was not yet long enough (HR 0.89; 95% CI 0.48 to 1.65). 

An overview of the observed adverse effects connected to therapy with the three TKIs is given in Table 2.

Many of the reported AEs can be very uncomfortable or even disabling for the affected patient, leading to loss of compliance or even discontinuation of the medication, but do not necessarily pose a severe health risk per se. Most of the common AEs like diarrhea, nausea, or fatigue can be relieved by measures such as dose adjustments, control of blood and electrolyte parameters, adjusting the patient’s daily schedule, and the additional administration of drugs such as loperamide. Hypertension, however, especially if left untreated, can lead to potentially life-threatening complications and should be controlled tightly over the course of the TKI therapy. In the pazopanib trial, 2.8% of treated patients had to stop treatment because of hypertension [22]. The assessment of the actual clinical importance of TKI-induced hypertension can be complex as a result of the association of different complications with hypertension in different age-groups, or inter-personal variations in how complications from hypertension affect quality of life. Nonetheless, it is a well-known fact that hypertension is associated with an increased risk of developing several high-mortality cardiovascular (CV) diseases, the three strongest associations being found with intracerebral hemorrhage, subarachnoid hemorrhage, and stable angina [43].

### 3.3. Management of Drug-Induced Hypertension

When making a management plan for the drug-induced hypertension, it is important to know if there are any interactions between the antihypertensive drugs and the chosen TKI. Both cabozantinib and pazopanib are metabolized by CYP3A4. Therefore, drugs altering the efficacy of this enzyme should be avoided in order to maintain the balance between the administered dose and the plasma clearance within the therapeutic interval [44,45]. 

One of the problems with TKI-induced hypertension is the lack of NO-production when blocking VEGF signaling. It is therefore imaginable that prescribing NO donors to hypertensive patients would induce a vasodilation, balancing the hypertensive side effects of the drugs. In a number of cases, the intake of donors, such as isosorbide dinitrate, isosorbide mononitrate, or molsidomine, has proven to significantly decrease hypertension, lowering the blood pressure to almost pre-treatment status [46,47]. Despite promising clinical efficacy and the development of a suggested treatment plan, the effect still needs to be evaluated in larger clinical trials [48].

As detailed above, the vasoconstrictor ET1’s may also be involved in the development of drug-induced hypertension as a result of overexpression. Endothelin receptor antagonists (ERAs) are already used for treating pulmonary arterial hypertension and have shown effects in treating systemic hypertension as well, but because of the side-effect complications such as liver toxicity and fluid retention, they are not the drug of choice for essential hypertension. However, it might find its clinical relevance in this case since the hypertension is TKI-induced [49,50].

Drug-induced hypertension is also treatable with more classic agents. Among these options, first-in-line therapies include angiotensin converting enzyme (ACE) inhibitors, angiotensin II receptor blockers, non-dihydropyridine calcium channel blockers, and beta-blockers. Some of these drugs are able to increase NO-signaling and based on the suggested positive effects of NO on hypertension, drugs with this ability, like the beta-blocker nevibolol, could be of particular interest. The two calcium-antagonists verapamil and diltiazem are both capable of inhibiting CYP3A4, and for this reason, they are interacting with the metabolism of pazopanib and cabozantinib. These antihypertensive drugs should therefore be excluded from consideration when managing the TKI-induced hypertension. 

Diarrhea is also an AE to TKI-treatment, which makes the electrolyte depleting drugs such as diuretics less important treatment options [51].

### 3.4. Ongoing Clinical Trials

There is still a need to validate cabozantinib, pazopanib, and vandetanib as a therapeutic option in MTC. Therefore, numerous clinical trials are currently investigating the efficacy, safety, and optimal doses of the drugs according to toxicity. An overview of currently ongoing trials is given in Table 3.

## 4. Discussion

A concern when treating any condition is that the efficacy of the chosen drug should always be sufficient enough to make up for the often rather extensive AEs. As specified above, hypertension is a common AE in treatments with TKIs [3], but also other serious AEs such as proteinuria, hemorrhage, pulmonary embolism/venous thrombosis, nausea, vomiting, diarrhea, and skin toxicity are of relevance and affect quality of life [26]. Hypertension as a side effect is not a problem, patients complain about it due to its asymptomatic nature. Therefore, it is not hypertension, but the cardiovascular complications following hypertension that are of concern and necessitate treatment [43].

A recent study investigated the predisposing factors for the development of hypertension during treatment with an anti-VEGF therapy like TKI. They found that pre-existing hypertension, an age of over 60 years, and a BMI above 25 are factors of significance. This should be kept in mind before starting treatment [54]. However, with the poor prognosis of a mean 10-year survival rate of 40% for progressive MTC [12], and with these drugs being administered in late stages of the disease, it raises the question whether hypertension is a severe concern, given that the TKIs are only life-prolonging by prevention of tumor growth—hence not curative—and only provide a somewhat short life extension. The median overall survival from treatment with pazopanib is approximately 20 months, and for treatments with cabozantinib between 20–45 months [22,43]. This relatively short treatment-period differentiates the problem with drug-induced hypertension from chronic essential hypertension, mainly because it is uncertain how long the patient has to suffer from hypertension in order to develop complications. Furthermore, antihypertensive drugs have AEs on their own, and by managing the drug-induced hypertension more AEs, or worsening of TKI-induced AEs, are likely to also affect the overall quality of life. Therefore, there is a fine line to walk. The caregiver has to personalize the treatment to each patient coming with a different health status both mentally and physically, and with different tolerance levels when it comes to the severity of AEs influencing the patient’s quality of life. Additionally, not treating hypertension in TKI-receiving patients could result in serious AEs such as cardiotoxicity worsening as a result of the high blood pressure. The treatment should therefore still be advised [55], making it a matter of finding the best treatment method. 

Interestingly, it was found that the overall survival was significantly longer amongst those patients developing hypertension. This could indicate that hypertension may be a biomarker for the drug’s efficacy [54], and it might therefore correlate positively with a greater benefit of TKI treatment. The risks of death from complications associated with hypertension are believed to be no greater than the risk of death from MTC itself, still shifting the risk-benefit-ratio in favor of the TKI treatment.

Tyrosine kinase inhibitors like pazopanib, cabozantinib, or vandetanib act by inhibiting the VEGF signaling cascade and have an anti-angiogenetic effect, preventing tumor growth and providing a promising treatment option to otherwise untreatable cancers. The drugs do not come without AEs though, the most common effect is hypertension. Hypertension is manageable in most cases, either through a lowering of TKI doses or through treatment with antihypertensive drugs. Unfortunately, in a few cases, the hypertension is so severe that the risk-benefit-ratio requires the discontinuation of TKI treatment. In all other cases, treatment with TKIs significantly prolongs progression-free survival for patients with progressive MTC. In addition, the treatment also prolongs overall survival, though not always to a statistically significant level. Therefore, developing treatment strategies for the AEs are required in order to secure TKI treatment for all patients with progressive MTC. 

At the moment, the process of finding the optimal dose for each patient is a delicate balance between still manageable AEs with an acceptable quality of life and a significant prolongation of PFS or OS. Finding suitable biomarkers for the prediction of TKI efficacy would therefore be an important contribution towards the optimization of personalized therapy. One promising candidate for this purpose is the *RET* mutation status. For vandetanib, however, benefits were observed for all patients, regardless of their *RET* status [28]. In the case of cabozantinib it was found that patients with a *RET* M918T mutation had the greatest benefit for PFS vs. placebo (R, 0.15; 95% CI, 0.08–0.28; *p*  < 0.0001) [56].

As demonstrated in the various clinical studies, the prevalence of AEs is still high and can in some cases lead to considerable rates of dose reduction or even termination of the treatment. It is therefore a major goal to deliver the drugs in a way that systemic AEs are minimized. One possibility for this are nanoscale drug delivery vehicles. In a recent study, a photoactivatable multi-inhibitor nanoliposome had been developed, which included cabozantinib. With the help of this contruct, it was possible to reduce tumor size and decrease metastasis after injection and near-infrared irradiaton of the tumor in a mouse pancreatic cancer model [57]. Furthermore, it was speculated that TKI-treatment might facilitate the delivery of liposme delivery via a so-called tumor normalization, but early experiments utilizing a mouse model of a lung cancer xenograft did not reveal any difference in intra-tumoral liposome concentration between pazopanib + and − mice [58].

## 5. Methods

As tools for finding the literature for this review, several online databases and registers were used, such as PubMed (https://www.ncbi.nlm.nih.gov/pubmed), Scopus (www.scopus.com), and ClinicalTrials (www.clinicaltrials.com). Articles and their references, only if written in English, were reviewed and those who were found relevant were included in this review. To limit the search in these databases, the following keywords were used: “Thyroid Cancer”, “Medullary Thyroid Cancer”, “Medullary Thyroid Cancer AND Pazopanib”, Medullary Thyroid Cancer AND Cabozantinib”, “Hypertension”, “Pazopanib AND hypertension”, “Cabozantinib AND Hypertension”, “Medullary Thyroid Cancer AND Vandetanib“, and lastly “Vandetanib AND hypertension”.

On 08/31/2018 a search in PubMed for “Thyroid Cancer” gave 71443 results, “Medullary Thyroid Cancer” gave 7550 results, “Medullary Thyroid Cancer AND Pazopanib” gave 14 results, “Medullary Thyroid Cancer AND Cabozantinib” gave 138 results, “Hypertension” gave 470284 results, “Pazopanib AND hypertension” gave 139 results, “Cabozantinib AND Hypertension” gave 36 results, Medullary Thyroid Cancer AND Vandetanib” gave 206 results, and lastly “Vandetanib AND Hypertension” gave 61 results.

## Figures and Tables

**Table 1:** Overview of compatible vascular endothelial growth factors (VEGFs) and VEGF receptors (VEGFRs).

VEGFR	VEGF
VEGFR-1	VEGF-A, VEGF-B and placental growth factors (PGF)
VEGFR-2	VEGF-A and proteolytically cleaved forms of VEGF-C and VEGF-D
VEGFR-3	VEGF-C and VEGF-D

**Table 2:** Adverse effects during tyrosine kinase inhibitor (TKI)-therapy of progressive medullary thyroid cancer (MTC).

Drug	Most Common Adverse Events (Any Grade)	Dose Reductions	Discontinuations Due to Aes
**Cabozantinib [34]**	Diarrhea (63%; 135/214) Hand-foot syndrome (50%; 107/214) Nausea (43%; 92/214) Fatigue (41%; 87/214) Hypertension (33%; 70/214)	79%; 169/214	16%; 35/214
**Pazopanib [22]**	Diarrhea (77%; 27/35) Fatigue (63%; 22/35) Hypertension (52%; 18/35) Nausea (52%; 18/35) Anorexia (46%; 16/35)	40%; 14/35	8.6%; 3/35
**Vandetanib [28]**	Diarrhea (56%; 130/231) Rash (45%; 104/231) Nausea (33%; 77/231) Hypertension (32%; 73/231) Headache (25%; 59/231)	35%; 81/231	12%; 28/231

Rates are given in %, followed by absolute numbers of affected patients/total patients.

**Table 3:** Overview of currently ongoing clinical trials studying pazopanib, cabozantinib, and vandetanib in MTC [52].

Drug	Title	Design	Objective	Status	Results
Pazopanib	A Phase II Study of GW 786034 (Pazopanib) in Advanced Thyroid Cancer NCT00625846	Interventional Open label	To establish safety and efficacy of pazopanib in Differentiated thyroid cancer (DTC), Medullary thyroid cancer (MTC), and Anaplastic thyroid cancer (ATC), how the drug impacts VEGF plasma levels and the changes is thyroglobulin and its relationship with tumor response.	Active, Not recruiting	The study found positive partial response rates in DTC, but has no overall survival (OS) measure. Adverse effects (AEs) occurred in 43% but generally mild. It showed to be a positive treatment option [53].
Cabozantinib	A Randomized, Double-blind Study To Evaluate the Efficacy and Safety of Cabozantinib (XL184) at 60 mg/Day Compared to a 140 mg/Day in Progressive, Metastatic Medullary Thyroid Cancer Patients NCT01896479	Interventional Double-blind Randomized	To study the efficacy and safety of cabozantinib when comparing to different doses: 60 mg and 140 mg with placebo. Efficacy measures are progression free survival and overall response rate. Safety measures is a comparison between fewer adverse effects and the efficacy measures.	Recruiting	−
	An Open-Label, Expanded Access Study of Cabozantinib (XL184) in Subjects With Unresectable, Locally Advanced, or Metastatic Medullary Thyroid Cancer NCT01683110	Expanded access Open Label	Provide access to cabozantinib	Approved for marketing	−
	An International, Randomized, Double-Blinded, Phase 3 Efficacy Study of XL184 Versus Placebo in Subjects With Unresectable, Locally Advanced, or Metastatic Medullary Thyroid Cancer NCT00704730	Interventional Double-blind Randomized	This study is comparing efficacy measures such as progression-free survival, overall survival and objective response rate between patients receiving cabozantinib and placebo.	Unknown, has results	The study found a statistically significant increase in Progression free survival (PFS) using cabozantinib compared to placebo, an objective response rate of 28% (higher for *RET* M918T positive subgroup) and an improved OS, although not significantly better than placebo [42,43].
	A Phase 1 Dose-Escalation Study of the Safety and Pharmacokinetics of XL184 Administered Orally to Subjects With Advanced Malignancies NCT00215605	Interventional Open label	This study is evaluating the safety, tolerability, maximum tolerated dose and dose-limiting toxicity of cabozantinib. It also evaluates plasma pharmacokinetics and renal elimination. Measurements include progression-free survival and tumor response.	Completed	49% of patients with MTC showed tumor shrinkage and 68% of these showed stable disease for over 6 months. Of all patients included, 90% experienced AEs, but 43% were of grade 1 or 2. The drug was considered to have a satisfactory safety profile [34]
	A Phase 1 Study of XL184 (Cabozantinib) in Children and Adolescents With Recurrent or Refractory Solid Tumors, Including CNS Tumors NCT01709435	Interventional Open label	It is evaluating maximum tolerated dose and recommended phase II dose of cabozantinib in children with solid tumors incl. childhood thyroid gland medullary carcinomas. They also describe toxicities, pharmacokinetics and evaluate overall survival.	Active, Not recruiting	−
	Phase 2 Trial of XL184 (Cabozantinib) an Oral Small-Molecule Inhibitor of Multiple Kinases, in Children and Young Adults With Refractory Sarcomas, Wilms Tumor, and Other Rare Tumors NCT02867592	Interventional, Open label	Determine the objective response rate, toxicities, and pharmacokinetics of cabozantinib in children and young adults with rare tumors.	Recruiting	
Vandetanib	European, Observational, Prospective Study to Evaluate the Benefit/Risk of Vandetanib in *RET* Mutation Negative and Positive Patients With Symptomatic, Aggressive, Sporadic, Unresectable, Locally Advanced/Metastatic Medullary Thyroid Cancer NCT01945762	Observational	Assessment of the risk/benefit of 300 mg/d vandetanibin *RET* positive and *RET* negative patients with MTC.	Recruiting	
	Phase I/II Trial of Vandetanib (ZD6474, ZACTIMA) in Children and Adolescents With Hereditary Medullary Thyroid Carcinoma NCT00514046	Interventional	Investigation of activity of vandetanib in children and adolescents with MTC caused by multiple endocrine neoplasia genetic disorder. Assessment of safety, tolerability, and survival in the study group.	Active, not recruiting	
	A Randomized, Int., Open-Label Phase III Study to Assess the Effect of a Patient Outreach Program on the Percentage of Time Patients With Locally Advanced or Metastatic MTC Experience Grade 2 or Higher AEs in the First 12 Months of Treatment With Vandetanib NCT01298323	Randomized, Open label	Determine if contacting patients with MTC more frequently results in earlier detection and treatment of signs and symptoms of AEs during the first 12 months on vandetanib treatment.	Active, not recruiting	
	An International, Randomised, Double-Blind, Two-Arm Study To Evaluate The Safety And Efficacy Of Vandetanib 150 And 300mg/Day In Patients With Unresectable Locally Advanced Or Metastatic Medullary Thyroid Carcinoma With Progressive Or Symptomatic Disease NCT01496313	Interventional, Randomized, Double-blind	Comparison of safety and efficacy of 150 and 300 mg/d vandetanib in patients with MTC.	Active, not recruiting	

## Abbreviations

AE(s)	Adverse effect(s)
AKT	Protein kinase
APWV	Aortic pulse wave velocity
ATC	Anaplastic thyroid cancer
BAD	BCL2 associated agonist of cell death
CAIx	Central aortic augmentation index
c-Kit	Stem cell growth factor receptor
CV	Cardiovascular
CYP3A4	Cytochrome P_450_3A4
DBP	Diastolic blood pressure
DTC	Differentiated thyroid cancer
EGF	Epidermal growth factor
ERA	Endothelin receptor antagonist
ERK	Extracelluar regulated kinase
FC	Follicular cell
FGF	Fibroblast growth factor
FMTC	Familial medullary thyroid cancer
FTC	follicular thyroid cancer
GF	Growth factors
HGF	Hepatocyte growth factor
MAPK	Mitogen activated protein kinase
MEK	Methyl ethyl ketone
MET	Methionine
MDM2	Mouse double minute 2
MTC	Medullary thyroid cancer
mTOR	Mammalian rarget of rapamycin
NF-kB	Nuclear factor kappa B
OS	Overall survival
PDGF	Platelet derived growth factor
PDGFR	Platelet derived growth factor receptor
PDTC	Poorly differentiated thyroid cancer
PFS	Progression free survival
PI3K	Phosphoinositide 3 kinases
PR	Partial response
PTC	Papillary thyroid cancer
RAF	Rapidly accelerated fibrosarcoma
RAS	Rat sarcoma
RECIST	Response Evaluation Criteria in Solid Tumors
RET	Rearranged during Transfection
RTK	Receptor tyrosine kinase
SBP	Systolic blood pressure
STAT	Signal transducers and activators of transcription
TK	Tyrosine kinase
TKI	Tyrosine kinase inhibitor(s)
VEGF	Vascular endothelial growth factor
VEGFR	Vascular endothelial growth factor receptor

## References

[1] Griebeler M.L., Gharib H., Thompson G.B. (2013). Medullary thyroid carcinoma. Endocr. Pract..

[2] Priya S.R., Dravid C.S., Digumarti R., Dandekar M. (2017). Targeted therapy for medullary thyroid cancer: A review. Front. Oncol..

[3] Kandula P., Agarwal R. (2011). Proteinuria and hypertension with tyrosine kinase inhibitors. Kidney Int..

[4] Cheah W.K. (2007). Thyroid cancer: Diagnosis and management. Singap. Med. J..

[5] Patel K.N., Shaha A.R. (2006). Poorly differentiated and anaplastic thyroid cancer. Cancer Control.

[6] Howlader N., Noone A.M., Krapcho M., Miller D., Bishop K., Altekruse S.F., Kosary C.L., Yu M., Ruhl J., Tatalovich Z. Seer Cancer Statistics Review, 1975–2013. https://seer.Cancer.Gov/csr/1975_2013/.

[7] Blomberg M., Feldt-Rasmussen U., Andersen K.K., Kjaer S.K. (2012). Thyroid cancer in Denmark 1943–2008, before and after iodine supplementation. Int. J. Cancer.

[8] Ferreira C.V., Siqueira D.R., Ceolin L., Maia A.L. (2013). Advanced medullary thyroid cancer: Pathophysiology and management. Cancer Manag. Res..

[9] Elisei R., Cosci B., Romei C., Bottici V., Renzini G., Molinaro E., Agate L., Vivaldi A., Faviana P., Basolo F. (2008). Prognostic significance of somatic ret oncogene mutations in sporadic medullary thyroid cancer: A 10-year follow-up study. J. Clin. Endocrinol. Metab..

[10] Quayle F.J., Moley J.F. (2005). Medullary thyroid carcinoma: Management of lymph node metastases. Curr. Treat. Opt. Oncol..

[11] Modigliani E., Cohen R., Campos J.M., Conte-Devolx B., Maes B., Boneu A., Schlumberger M., Bigorgne J.C., Dumontier P., Leclerc L. (1998). Prognostic factors for survival and for biochemical cure in medullary thyroid carcinoma: Results in 899 patients. The getc study group. Groupe d’etude des tumeurs a calcitonine. Clin. Endocrinol..

[12] Roman S., Lin R., Sosa J.A. (2006). Prognosis of medullary thyroid carcinoma: Demographic, clinical, and pathologic predictors of survival in 1252 cases. Cancer.

[13] Massicotte M.H., Brassard M., Claude-Desroches M., Borget I., Bonichon F., Giraudet A.L., Do Cao C., Chougnet C.N., Leboulleux S., Baudin E. (2014). Tyrosine kinase inhibitor treatments in patients with metastatic thyroid carcinomas: A retrospective study of the tuthyref network. Eur. J. Endocrinol..

[14] Schlessinger J. (2000). Cell signaling by receptor tyrosine kinases. Cell.

[15] Arora A., Scholar E.M. (2005). Role of tyrosine kinase inhibitors in cancer therapy. J. Pharmacol. Exp. Ther..

[16] Dvorak H.F. (2005). Angiogenesis: Update 2005. J. Thromb. Haemost..

[17] Ferrara N. (2004). Vascular endothelial growth factor: Basic science and clinical progress. Endocr. Rev..

[18] Kowanetz M., Ferrara N. (2006). Vascular endothelial growth factor signaling pathways: Therapeutic perspective. Clin. Cancer Res..

[19] Presta L.G., Chen H., O’Connor S.J., Chisholm V., Meng Y.G., Krummen L., Winkler M., Ferrara N. (1997). Humanization of an anti-vascular endothelial growth factor monoclonal antibody for the therapy of solid tumors and other disorders. Cancer Res..

[20] Yakes F.M., Chen J., Tan J., Yamaguchi K., Shi Y., Yu P., Qian F., Chu F., Bentzien F., Cancilla B. (2011). Cabozantinib (xl184), a novel met and vegfr2 inhibitor, simultaneously suppresses metastasis, angiogenesis, and tumor growth. Mol. Cancer Ther..

[21] Kurzrock R., Sherman S.I., Ball D.W., Forastiere A.A., Cohen R.B., Mehra R., Pfister D.G., Cohen E.E., Janisch L., Nauling F. (2011). Activity of xl184 (cabozantinib), an oral tyrosine kinase inhibitor, in patients with medullary thyroid cancer. J. Clin. Oncol..

[22] Bible K.C., Suman V.J., Molina J.R., Smallridge R.C., Maples W.J., Menefee M.E., Rubin J., Karlin N., Sideras K., Morris I.I.I.J.C. (2014). A multicenter phase 2 trial of pazopanib in metastatic and progressive medullary thyroid carcinoma: Mc057h. J. Clin. Endocrinol. Metab..

[23] Kumar R., Knick V.B., Rudolph S.K., Johnson J.H., Crosby R.M., Crouthamel M.C., Hopper T.M., Miller C.G., Harrington L.E., Onori J.A. (2007). Pharmacokinetic-pharmacodynamic correlation from mouse to human with pazopanib, a multikinase angiogenesis inhibitor with potent antitumor and antiangiogenic activity. Mol. Cancer Ther..

[24] Karras S., Pontikides N., Krassas G.E. (2013). Pharmacokinetic evaluation of cabozantinib for the treatment of thyroid cancer. Expert Opin. Drug Metab. Toxicol..

[25] Verheijen R.B., Beijnen J.H., Schellens J.H.M., Huitema A.D.R., Steeghs N. (2017). Clinical Pharmacokinetics and Pharmacodynamics of Pazopanib: Towards Optimized Dosing. Clin. Pharmacokinet..

[26] Carlomagno F., Vitagliano D., Guida T., Ciardiello F., Tortora G., Vecchio G., Ryan A.J., Fontanini G., Fusco A., Santoro M. (2002). Zd6474, an orally available inhibitor of kdr tyrosine kinase activity, efficiently blocks oncogenic ret kinases. Cancer Res..

[27] Wedge S.R., Ogilvie D.J., Dukes M., Kendrew J., Chester R., Jackson J.A., Boffey S.J., Valentine P.J., Curwen J.O., Musgrove H.L. (2002). Zd6474 inhibits vascular endothelial growth factor signaling, angiogenesis, and tumor growth following oral administration. Cancer Res..

[28] Wells S.A., Gosnell J.E., Gagel R.F., Moley J., Pfister D., Sosa J.A., Skinner M., Krebs A., Vasselli J., Schlumberger M. (2010). Vandetanib for the treatment of patients with locally advanced or metastatic hereditary medullary thyroid cancer. J. Clin. Oncol..

[29] Robinson B.G., Paz-Ares L., Krebs A., Vasselli J., Haddad R. (2010). Vandetanib (100 mg) in patients with locally advanced or metastatic hereditary medullary thyroid cancer. J. Clin. Endocrinol. Metab..

[30] Wells S.A., Robinson B.G., Gagel R.F., Dralle H., Fagin J.A., Santoro M., Baudin E., Elisei R., Jarzab B., Vasselli J.R. (2012). Vandetanib in patients with locally advanced or metastatic medullary thyroid cancer: A randomized, double-blind phase iii trial. J. Clin. Oncol..

[31] Fox E., Widemann B.C., Chuk M.K., Marcus L., Aikin A., Whitcomb P.O., Merino M.J., Lodish M., Dombi E., Steinberg S.M. (2013). Vandetanib in children and adolescents with multiple endocrine neoplasia type 2b associated medullary thyroid carcinoma. Clin. Cancer Res..

[32] Frampton J.E. (2012). Vandetanib: In medullary thyroid cancer. Drugs.

[33] Resteghini C., Cavalieri S., Galbiati D., Granata R., Alfieri S., Bergamini C., Bossi P., Licitra L., Locati L.D. (2017). Management of tyrosine kinase inhibitors (tki) side effects in differentiated and medullary thyroid cancer patients. Best Pract. Res. Clin. Endocrinol. Metab..

[34] Elisei R., Schlumberger M.J., Müller S.P., Schöffski P., Brose M.S., Shah M.H., Licitra L., Jarzab B., Medvedev V., Kreissl M.C. (2013). Cabozantinib in progressive medullary thyroid cancer. J. Clin. Oncol..

[35] Cabanillas M.E., Hu M.I., Durand J.B., Busaidy N.L. (2011). Challenges associated with tyrosine kinase inhibitor therapy for metastatic thyroid cancer. J. Thyroid Res..

[36] Hoy S.M. (2014). Cabozantinib: A review of its use in patients with medullary thyroid cancer. Drugs.

[37] Facemire C.S., Nixon A.B., Griffiths R., Hurwitz H., Coffman T.M. (2009). Vascular endothelial growth factor receptor 2 controls blood pressure by regulating nitric oxide synthase expression. Hypertension.

[38] Horowitz J.R., Rivard A., van der Zee R., Hariawala M., Sheriff D.D., Esakof D.D., Chaudhry G.M., Symes J.F., Isner J.M. (1997). Vascular endothelial growth factor/vascular permeability factor produces nitric oxide-dependent hypotension. Evidence for a maintenance role in quiescent adult endothelium. Arterioscler. Thromb. Vasc. Biol..

[39] Steeghs N., Gelderblom H., Roodt J.O., Christensen O., Rajagopalan P., Hovens M., Putter H., Rabelink T.J., de Koning E. (2008). Hypertension and rarefaction during treatment with telatinib, a small molecule angiogenesis inhibitor. Clin. Cancer Res..

[40] Matsuura A., Yamochi W., Hirata K., Kawashima S., Yokoyama M. (1998). Stimulatory interaction between vascular endothelial growth factor and endothelin-1 on each gene expression. Hypertension.

[41] Veronese M.L., Mosenkis A., Flaherty K.T., Gallagher M., Stevenson J.P., Townsend R.R., O’Dwyer P.J. (2006). Mechanisms of hypertension associated with bay 43-9006. J. Clin. Oncol..

[42] Schlumberger M., Elisei R., Müller S., Schöffski P., Brose M., Shah M., Licitra L., Krajewska J., Kreissl M.C., Niederle B. (2017). Overall survival analysis of exam, a phase iii trial of cabozantinib in patients with radiographically progressive medullary thyroid carcinoma. Ann. Oncol..

[43] Rapsomaniki E., Timmis A., George J., Pujades-Rodriguez M., Shah A.D., Denaxas S., White I.R., Caulfield M.J., Deanfield J.E., Smeeth L. (2014). Blood pressure and incidence of twelve cardiovascular diseases: Lifetime risks, healthy life-years lost, and age-specific associations in 1·25 million people. Lancet.

[44] Nguyen L., Holland J., Miles D., Engel C., Benrimoh N., O’Reilly T., Lacy S. (2015). Pharmacokinetic (pk) drug interaction studies of cabozantinib: Effect of cyp3a inducer rifampin and inhibitor ketoconazole on cabozantinib plasma pk and effect of cabozantinib on cyp2c8 probe substrate rosiglitazone plasma pk. J. Clin. Pharmacol..

[45] Tan A.R., Gibbon D.G., Stein M.N., Lindquist D., Edenfield J.W., Martin J.C., Gregory C., Suttle A.B., Tada H., Botbyl J. (2013). Effects of ketoconazole and esomeprazole on the pharmacokinetics of pazopanib in patients with solid tumors. Cancer Chemother. Pharmacol..

[46] Kruzliak P., Kovacova G., Pechanova O. (2013). Therapeutic potential of nitric oxide donors in the prevention and treatment of angiogenesis-inhibitor-induced hypertension. Angiogenesis.

[47] Dirix L.Y., Maes H., Sweldens C. (2007). Treatment of arterial hypertension (aht) associated with angiogenesis inhibitors. Ann. Oncol..

[48] Kružliak P., Novák J., Novák M. (2014). Vascular endothelial growth factor inhibitor–induced hypertension: From pathophysiology to prevention and treatment based on long-acting nitric oxide donors. Am. J. Hypertens..

[49] Miyagawa K., Emoto N. (2014). Current state of endothelin receptor antagonism in hypertension and pulmonary hypertension. Ther. Adv. Cardiovasc. Dis..

[50] Laffin L.J., Bakris G.L. (2015). Endothelin antagonism and hypertension: An evolving target. Semin. Nephrol..

[51] Zamorano J.L., Lancellotti P., Rodriguez Muñoz D., Aboyans V., Asteggiano R., Galderisi M., Habib G., Lenihan D.J., Lip G.Y.H., Lyon A.R. (2016). 2016 esc position paper on cancer treatments and cardiovascular toxicity developed under the auspices of the esc committee for practice guidelines the task force for cancer treatments and cardiovascular toxicity of the european society of cardiology (esc). Eur. Heart J..

[52] ClinicalTrials.gov Is a Database of Privately and Publicly Funded Clinical Studies Conducted around the World. http://www.clinicaltrials.gov.

[53] Bible K.C., Suman V.J., Molina J.R., Smallridge R.C., Maples W.J., Menefee M.E., Rubin J., Sideras K., Morris J.C., McIver B. (2010). Efficacy of pazopanib in progressive, radioiodine-refractory, metastatic differentiated thyroid cancers: Results of a phase 2 consortium study. Lancet Oncol..

[54] Hamnvik O.P., Choueiri T.K., Turchin A., McKay R.R., Goyal L., Davis M., Kaymakcalan M.D., Williams J.S. (2015). Clinical risk factors for the development of hypertension in patients treated with inhibitors of the vegf signaling pathway. Cancer.

[55] Maitland M.L., Bakris G.L., Black H.R., Chen H.X., Durand J.B., Elliott W.J., Ivy S.P., Leier C.V., Lindenfeld J., Liu G. (2010). Initial assessment, surveillance, and management of blood pressure in patients receiving vascular endothelial growth factor signaling pathway inhibitors. J. Natl. Cancer Inst..

[56] Sherman S.I., Clary Clary, D.O., Elisei R., Schlumberger M.J., Cohen E.E., Schöffski P., Wirth L.J., Mangeshkar M., Aftab D.T., Brose M.S. (2016). Correlative analyses of RET and RAS mutations in a phase 3 trial of cabozantinib in patients with progressive, metastatic medullary thyroid cancer. Cancer.

[57] Spring B.Q., Bryan Sears R., Zheng L.Z., Mai Z., Watanabe R., Sherwood M.E., Schoenfeld D.A., Pogue B.W., Pereira S.P., Villa E. (2016). A photoactivable multi-inhibitor nanoliposome for tumour control and simultaneous inhibition of treatment escape pathways. Nat. Nanotechnol..

[58] Tailor T.D., Hanna G., Yarmolenko P.S., Dreher M.R., Betof A.S., Nixon A.B., Spasojevic I., Dewhirst M.W. (2010). Effect of pazopanib on tumor microenvironment and liposome delivery. Mol. Cancer Ther..

